# *Streptococcus equi subsp. zooepidemicus* and ‘Neighbourhood syndrome’ –extra-orbitocranial rhinosinusitis with reversible sudden loss of vision- a case report

**DOI:** 10.1186/s12886-019-1126-x

**Published:** 2019-05-20

**Authors:** John Kutsukutsa, Nthabeleng Rankhethoa, Jaivani Sharvani Pillay, Johannes Frederik De Jager, Zaynah Dangor, Yesholata Mahabeer

**Affiliations:** 1 Department of Otorhinolaryngology Head & Neck Surgery, University of KwaZulu-Natal, Durban, 4001 South Africa; 2 Department of Radiology, Inkosi Albert Luthuli Central Hospital, Mayville, Durban, 4058 South Africa; 3 Department of Opthalmology, University of KwaZulu-Natal, Durban, 4001 South Africa; 4 Department of Neurology, University of KwaZulu-Natal, Durban, 4001 South Africa; 5 Department of Medical microbiology, University of KwaZulu-Natal and National Health Laboratory Services, Durban, South Africa

**Keywords:** Rhinosinusitis, Loss of vision, *Streptococcus zooepidermicus*

## Abstract

**Background:**

Rhinosinusitis is a common condition which may present with complications commonly involving the orbit and the intracranial space. Loss of vision in the absence of clinical or radiological signs of involvement of the orbit and intracranium is rare and carries a high morbidity rate. *Streptococcus equi subspecies zooepidemicus* is not a well-documented cause of paranasal sinus infection.

**Case presentation:**

We present a case of a 16 year old female patient who had unilateral loss of vision with signs of retrobulbar optic neuritis and no other neurological signs. We isolated an unusual organism- *Streptococcus equi subspecies zooepidemicus* from the maxillary sinus. Emergency endoscopic sinus surgery and antibiotic treatment resulted in complete reversal of the loss of vision.

**Conclusion:**

The presence of paranasal sinus disease in association with loss of vision even in the absence of a clear link between the two should be treated as an emergency with surgical drainage and or appropriate antibiotic therapy. In patients presenting with suspected inflammatory orbital involvement, imaging of the orbit and paranasal sinuses should be considered early.

## Background

The pathophysiology of intra-orbital and intracranial complications of rhinosinusitis has been well-described [1–5]. Loss of vision is an uncommon finding which is more frequently a complication of intra-orbital involvement (abscess or cellulitis) or less commonly of well described syndromes, the orbital apex syndrome(OAS) and carvernous sinus syndrome (CSS) [6]. OAS describes as a syndrome involving damage to the oculomotor, trochlea, abducens nerves, and ophthalmic branch of the trigeminal nerve in association with optic nerve dysfunction. CSS is the involvement of the maxillary branch of the trigeminal nerve and occulosympathetic fibres in addition to features of OAS [7].

The common pathogens causing community acquired acute rhinosinusitis are *Streptococcus pneumoniae, Hemophilus influenzae, Moraxella catarhalis* while those implicated in chronic rhinosinusitis are staphylococci, enteric gram negative bacilli and anaerobes [8–11]*. Streptococcus equi subsp. zooepidemicus* a Lancefield Group C streptococcus, is a commensal or pathogen in a horses, cattle, swine and guinea pigs [12, 13]. In humans, infections caused by *Streptococcus equi subsp. zooepidemicus* are uncommon with a variety of pyogenic clinical manifestations; skin and soft tissue infections, septic arthritis, endocarditis, meningitis, pneumonia,, pharyngitis, lymphadenitis and osteomyelitis [13–16]. Human infection is considered a zoonosis generally associated with contact with horses, and consumption of unpasteurized milk products, goat cheese or pork [12, 16].The true incidence of *Streptococcus equi subsp. zooepidemicus* infections are not known and probably under reported because few laboratories routinely determine species [13, 17]. It is a highly virulent organisms sharing 80% sequence homology with the known *Streptococcus pyogenes*, with many virulence factors in common [15, 18]. The usual entry routes of S*. equi subsp. zooepidemicus* in humans are the respiratory or gastrointestinal tracts and skin [16]. To the best of our knowledge, this organism has not been described as a cause of rhinosinusitis with associated loss of vision.

‘Neighbourhood syndrome’ is a diagnosis of exclusion used to describe inflammatory involvement of related structures.

We present a patient with rhino sinusitis complicated by reversible loss of vision with no identifiable radiological orbitocranial extension or optic canal bony defect in whom *Streptococcus equi subspecies zooepidemicus* was cultured. We aim to describe the unusual case presentation and management with a review of literature on loss of vision in sinusitis.

## Case presentation

A 16-year-old, black female patient was referred from the ophthalmology service to our Ear Nose and Throat (ENT) unit at Inkosi Albert Luthuli Central Hospital, Durban South Africa with acute progressive right sided loss of vision for 3 days. It was associated with intermittent ipsilateral moderate-intensity frontal headaches radiating to the ipsilateral temporal area. She had been diagnosed with optic neuritis and commenced on methylprednisolone (250 mg intravenously every 6 h) 2 days previously, with minimal improvement. She noted disturbance in colour vision, and pain on eye movement but no diplopia prior to loss of vision. Computed tomographic (CT) imaging had shown pan sinusitis resulting in her referral to the ENT unit. She had chronic rhinosinusitis for which she was on treatment with a steroid spray and nasal douche. She did not have current or recent nasal obstruction, purulent nasal discharge or facial pressure. There was no associated fever, nausea or vomiting, no history of trauma, or preceding acute illness (particularly no recent upper respiratory tract infection), no other neurological symptoms or any periorbital swelling. She was a student in a metropolitan high school, did not smoke or consume alcohol, and denied any contact with animals or consumption of unpasteurised milk. She reported visiting her rural home approximately 6 weeks prior to presentation but denied consuming any raw milk or contact with horses. Her last normal menstrual cycle was a week prior to presentation.

On examination, she was a well looking patient, who was not acutely ill, fully conscious and had normal vital signs. ENT examination was normal except for an inflamed nasal mucosa. She had poor light perception on the right side (progressively worse since the initial visual acuity of counting fingers at first presentation to ophthalmology) and decreased colour appreciation on Ishihara chart assessment. She had full range of extra ocular motility and anterior segment examination was normal. Posterior segment examination revealed quiet vitreous, no papilledema or optic disc erythema; there were no retinal or macula abnormalities. There was no relative afferent pupillary defect noted. Left eye examination was normal with a visual acuity of 6/12 (20/40). She had no signs of meningism and the rest of cranial nerve examination was normal. The rest of the systems examination was normal.

**Fig. 1:**
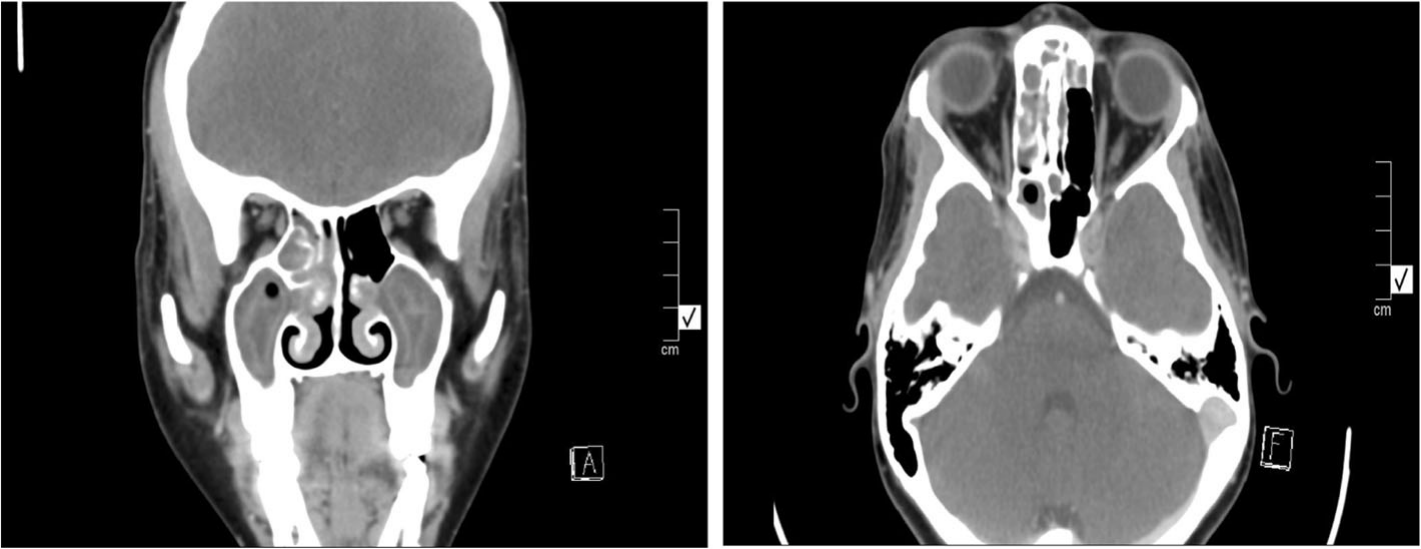
Coronal and axial contrast-enhaced CT demonstrates bilateral maxillary sinus, right ethmoidal, frontal and sphenoidal opacification. The superior ophthalmic veins were bilaterally enhancing with no filling defects. There was no caroto-cavernous sinus pathological enhancement seen. There were no retro-bulbar lesions noted or inflammatory changes noted

**Fig. 2:**
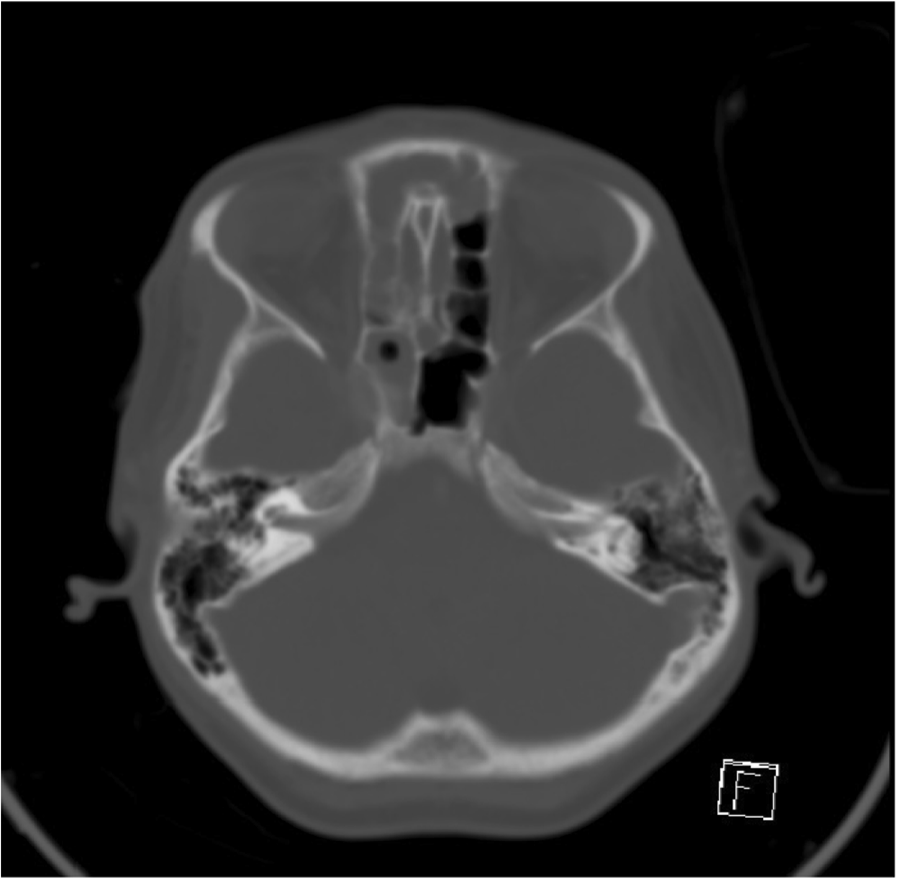
Axial CECT on bone window settings demonstrates no evidence of bony destruction or unilateral widening of the optic canal. Pansinusitis noted

Full blood count, urea and electrolytes and Erythrocyte sedimentation rate was normal. Rapid Plasma Reagin for syphilis was negative, C-reactive protein was less than 10 mg/L and a rapid HIV test was negative. Coagulation studies were normal and D-dimer was negative. Computed Tomographic (CT) scan (Fig. 1) showed bilateral maxillary sinus, right ethmoidal, frontal and sphenoidal opacification. The superior ophthalmic veins were bilaterally enhancing with no filling defects. There was no caroto-cavernous sinus pathological enhancement seen. There were no retro-bulbar lesions noted, no orbital or pre-septal orbital cellulitis. There were no intracranial lesions noted, no ipsilateral bony defects of lamina papyracea, optic canal or lateral sphenoidal wall noted (Fig. 2). There were no bony lesions noted in relation to the skull base in the midline.

There was no obvious direct link between loss of vision and the sinusitis, nevertheless a decision was taken to start the patient empirically on intravenous Amoxicillin/ Clavulanate (1.2 g three times a day) and perform emergency functional endoscopic sinus surgery. She was taken to theatre within 24 h of admission to the ENT ward (approximately 5 days since onset of loss of vision) where she had bilateral middle meatus antrostomy with maxillary sinus washout, right total ethmoidectomy, right sphenoidotomy and right frontal sinusotomy. Pus was found in both maxillary sinuses as well as in the anterior and posterior ethmoid sinuses on the right side, the sphenoid sinus had oedematous mucosa. There was oedematous mucosa in the left sphenoid and frontal sinuses with no pus. The right side was packed with Merocel® (Medtronic Xomed Inc) postoperatively.

Nasal pack was removed on day 1 postoperatively and nasal douche as well as steroid spray was commenced. She completed a 3 day methylprednisone course and continued on intravenous Amoxicillin/Clavulanate. She had no periorbital swelling and remained stable. The patient reported an improvement in vision day 1 post operatively.

Intraoperative swabs taken from maxillary sinus revealed *Streptococcus equi subsp. zooepidemicus* susceptible to Amoxicillin-Clavulanate. This was reported as a moderate growth on culture and was also picked up on microscopy. Specific identification was done by Lancefield grouping as well as the automated Vitek® 2 system. Histology of intra operative sinus tissue reported that the features were in keeping with chronic allergic sinusitis; no fungi demonstrated, no features of an osteitis, no granulomas or viral inclusions and no tumor. She had progressive subjective improvement in vision and had normal eye examination findings with a visual acuity of 6/9 (20/30) in both eyes on discharge, 4 days postoperatively. She was reviewed by a neurologist pre- and post-operatively and diagnosed with ‘neighbourhood syndrome’. Further imaging was deferred given the marked and complete recovery post surgery and antibiotics. She was discharged home on oral Amoxicillin/Clavulanate to be continued for another 10 days and long term nasal douche and Fluticasone nasal spray.

Two week and 4 week follow-ups were unremarkable; vision was still maintained and nasal mucosa inflammation had subsided.

## Discussion and Conclusion

Rhinosinusitis is a well know pathological entity. Despite some grey areas in its pathophysiology and management, there is some consensus on its aetiology, classification, and management [8, 9, 11, 19, 20]. It is recognized to cause orbital and intracranial complications ranging from the benign preseptal cellulitis to the severe intracranial infections [1, 4, 5, 21]. The involvement of the orbit and intracranial space is linked to their intimate anatomical relationship with the paranasal sinuses. The nasal and paranasal sinus valveless venous drainage through orbit into intracranial sinuses also contributes to this process [1]. It is generally accepted that orbital involvement is heralded by periorbital or orbital symptoms such as swelling, proptosis, diplopia and those of the intracranium by neurological signs. These were nonexistent in our patient.

The differential diagnosis for loss of vision is quite extensive. Vision involves an image passing through the refractive structures of the eye to reach the retina where the sensory cells are, onto the optic nerve out into orbit, orbital apex, intracranial space onto the occipital lobe of the brain for processing. Problems anywhere along that pathway can lead to loss of vision. Visual loss is an uncommon but recognized complication of bacterial sinusitis. The mechanisms by which this may occur are several-fold:

- Direct spread of the sinus infection to the optic nerve [22, 23]
- elevation of the intraorbital pressure resulting in retinal ischemia [24]
- optic neuritis due to the spread of infection [23–25]
- Osteomyelitis of the sinus walls (which may be in direct contact with the nerve)
- Ethmoid or sphenoid sinus mucoceles or mucopyoceles [23, 25]
- Polyps involving the mucosa of the sphenoid sinus [23]
- Rhinogenic optic neuropathy, where optic nerve dysfunction occurs as a result of infection or mucocoeles of the Onodi (spheno-ethmoidal) cell. Its proximity to the optic nerve results in contiguous spread and subsequent visual loss [25]
- Venous occlusion resulting in nerve ischaemia [22]
- Central retinal artery occlusion [4]
- Oedema due to an inflammatory response resulting in nerve ischaemia [4]
- An immune response initiated by the infectious or inflammatory process within the sinuses [23]

Our patient had signs of retrobulbar optic neuritis, an otherwise normal slit lamp examination and no other craniopathies. The conclusion was that of an isolated optic nerve pathology. The relative afferent pupillary defect (RAPD) tested by shining light into the eyes and checking their pupillary response can be useful for detecting unilateral or asymmetrical disease of the retina or optic nerve (but only optic nerve disease that occurs in front of the optic chiasm) [26]. A positive RAPD may be found with ischaemic optic neuropathy, optic neuritis, optic nerve compression (orbital tumours or dysthyroid eye disease), trauma, and asymmetric glaucoma and less commonly with infective, infiltrative, carcinomatous, or radiation optic neuropathy [26]. It has also however been noted in one animal study that neuronal loss of 25–50% is required to produce a positive RAPD [27]. In our patient we expected a positive RAPD as we often do especially with visual acuity of light perception but the converse was found. This may possibly be attributed to human factors which may influence identification and appropriate quantification of RAPD. An inter examiner disagreement of as high as 39% has been documented before giving credence in our case that a positive RAPD may have been missed [28].

The presence of eye pain, loss of colour vision and reduced visual acuity suggests a diagnosis of optic neuritis in this patient. Her vision recovered remarkably following sinus drainage. In view that the margins of both superior orbital veins were found to be enhancing, we infer that this was consequent to an inflammatory process.

MRI is customarily the imaging modality of choice in optic neuritis; however, CT of the orbits and sinuses is the preferred baseline investigation in patients with visual loss and sinus disease, as it is superior for reviewing bony involvement, is able to determine whether there is intra-orbital extension of sinonasal disease in the ventral two-thirds of the orbit and can be used as an anatomic guide for endoscopic sinus surgery [23].

Following surgery, resolution of both the infection and resultant inflammation prompted good visual recovery in our patient. In the absence of radiological evidence of abscess formation, orbital inflammation and filling defects, we conclude that the optic neuritis in this patient was due to ‘neighbourhood syndrome,’ which refers to contiguous spread and associated inflammation.

Neighbourhood syndrome is a diagnosis of exclusion which in some cases like ours is not explored further to find definitive causes given clinical response.

The finding of the zoonotic organism *S.equi subsp. zooepidermicus* in the sinuses is unusual and most likely occurred following colonisation of the nose and paranasal sinuses prior to infection. Very little is known about the risk factors, presenting features and outcome, a situation compound by its various presentations [29]. Our patient had no obvious history of consumption of unpasteurized milk or contact with animals which are the usual modes of spread. *S. zooepidermicus* has been linked to a heterogenous variety of conditions because of hematogenous spread. However this is unlikely in our patient because of lack of signs and symptoms of severe infection which is usually occurs with bacteraemia. It is possible that other causes of the loss of vision like septic emboli with ophthalmic or central retinal artery occlusion may have occurred secondary to bacterial endocarditis [30]. It is important to be aware of this zoonosis as it is potentially preventable at the primary healthcare level through health education on unprocessed animal products.

The treatment of choice for *S.equi susbsp. Zooepidermicus* is penicillin although it is susceptible to a wide range of antimicrobials including cephalosporins, aminoglycosides, vancomycin and macrolides [16, 18, 29, 31, 32]. The duration of treatment is not entirely clear, however 14 days has been the minimum in reported cases of meningitis [17, 31]. Our patient had potential source control through surgery followed by antibiotics for 14 days with complete resolution.

Loss of vision carries with it high burden to both the patient and society and is potentially reversible if it is managed early and appropriately. The presence of paranasal sinus disease in association with loss of vision even in the absence of a clear link should be treated as an emergency with consideration of surgical drainage and appropriate antibiotic therapy. Clinicians should have a high index of suspicion in patients with history of sinus disease or sinus symptoms presenting with inflammatory eye conditions and should consider imaging early. Unexplained loss of vision with a background of sinus disease even in the absence of signs and symptoms of active sinus disease may warrant emergency imaging and surgery as dictated by the disease. The zoonotic *Streptococcus equi subsp. zooepidermicus* has been associated with unusual disease presentations that are usually severe. Given the growing number of case reports on this pathogen, laboratories should identify the subspecies level in cases Group C Streptococci to allow further characterisation of this infection.

## Abbreviations

CSS: Carvernous sinus syndrome
CT: Computed tomographic scan
ENT: Ear, Nose and Throat
HIV: Human Immunodeficiency Virus
MRI: Magnetic Resonce Imaging
OAS: Orbital apex syndrome
RAPD: Relative afferent pupillary defect
